# Repeated Pressure and Shear Stress at the Posterior Heel Following Localized Skin Cooling: Protocol for a Repeated Measures Cohort Study

**DOI:** 10.2196/73250

**Published:** 2025-07-21

**Authors:** Ralph Gordon, Charlotte Stevens, Peter Worsley, Davide Filingeri

**Affiliations:** 1 School of Health Sciences Faculty of Environmental Life Sciences University of Southampton Southampton United Kingdom

**Keywords:** pressure ulcer, wounds, temperature, local cooling, shear stress

## Abstract

**Background:**

Pressure in combination with shear forces can deform soft tissues and lead to development of pressure ulcers. The prevalence rate of pressure ulcers in the United Kingdom remains unacceptably high and can occur across the human lifespan. The posterior heel represents a common anatomical site for pressure ulcers due to soft tissues lying adjacent to bony prominences and being exposed to pressure and shear during lying postures. Localized cooling and interface materials that reduce shear may offer potentially therapeutic benefits in the development of pressure ulcers. Yet the physiological mechanisms underpinning the potential benefits of localized cooling are not fully understood.

**Objective:**

This study protocol aims to investigate how localized cooling influences the skin’s microvascular, inflammatory, structural, and perceptual tolerance to repeated shear loading at the heel.

**Methods:**

The protocol will be tested on individuals of different age, sex, skin tone, and comorbidities, using a repeated measures design. Three cohorts will be recruited: (1) young and healthy (n=35), (2) older and healthy (n=35), and (3) with spinal cord injury (n=35). Participants will complete 3 testing sessions using a custom-built shearing rig with integrated thermal plate, during which the posterior aspect of the heel will be exposed to a standardized mechanical stimulus to elicit repeated pressure and shear loading. The experimental condition of each session will be determined by the temperature of the thermal plate, which will be set to either 36 °C (no cooling), 24 °C (mild cooling), or 16 °C (strong cooling). Continuous measurements will include kinetic coefficient of friction (CoF) and skin blood flow (via laser Doppler flowmetry; 40 Hz). Pro- and anti-inflammatory biomarkers in skin sebum (via Sebutape), structural skin properties (via optical coherence tomography), skin conductance (in microsiemens) and ratings of thermal sensation, comfort, and acceptance (via Likert scales) will also be assessed before and after the shear stress protocol.

**Results:**

Recruitment began in January 2024. As of February 2025, 43 participants had been enrolled in the study. Data collection and analysis are ongoing, and published findings are expected to be available in early 2026.

**Conclusions:**

This analysis will help identify mechanisms of skin damage following repeated shear stress at the heel, furthering our understanding of superficial pressure ulcers. It will also establish physiological and perceptual thresholds for the protective effects of cooling from shearing-induced damage at the heel.

**International Registered Report Identifier (IRRID):**

DERR1-10.2196/73250

## Introduction

Pressure ulcers constitute a type of localized damage to the skin and/or underlying soft tissue resulting from prolonged periods of pressure or pressure in combination with shear forces [1]. In the United Kingdom alone, the annual cost of treating chronic wounds, including pressure ulcers, has been estimated to be approximately £8.3 billion (US $12.7 billion) [2]. Accordingly, an improved understanding of the fundamental mechanisms underlying the physiological tolerance of human skin to mechanical loading and shearing could lead to the development of cost-effective, personalized solutions to prevent these wounds and improve patient care and quality of life [3].

Sustained localized mechanical loading and shearing of the skin can arise from lying and sitting postures, particularly in individuals with mobility impairment such as spinal cord injury (SCI) [4]. Internal tissue deformations occur as a result of sustained pressure and shear forces that can lead to changes in the physiology of skin and subdermal tissue, including ischemia in the blood vasculature, lymphatic impairment, and direct deformation damage [5,6]. It has been reported that nearly 30% of pressure ulcers occur at the heel, with this region being the second most likely area for pressure damage after the sacrum [7,8]. As opposed to the thick skin of the plantar heel, the posterior heel has thin, striated skin [9], which is very susceptible to pressure- and shear-induced damage [10]. Yet the mechanisms underlying the elevated risk of shear-induced damage at the heel remain under-studied.

Microclimate conditions within and around skin tissues strongly influence the tolerance of the skin to mechanical loading and shear [11]. For example, elevated temperature and humidity at the skin interface reduce the mechanical stiffness and strength of the skin [12] while also increasing its coefficient of friction [13]. These mechanisms can lead to a greater risk of tissue damage for the same mechanical stress [14,15]. In contrast, cooling reduces skin tissue’s metabolic demands and could increase the skin’s physiological tolerance to mechanical loads [12,16]. Early animal studies revealed that reduced skin temperature minimizes the risk of pressure ulcer formation [17,18]. This may occur through altered microvascular responses [18], as well as via downregulation of the expression of pro-inflammatory cytokines such as tumor necrosis factor α (TNFα), which is mediated by the stimulation of cold-sensitive transient receptor potential melastatin 8 (TRPM8)–expressing neurons in dorsal root ganglia [19,20]. Furthermore, our preliminary proof-of-concept experimental and modeling data on a representative skin site (the finger) indicated that skin cooling to 16 ºC can reduce friction by up to 35% for the same mechanical load [13]. This effect is likely due to changes in the viscoelastic properties of cooled skin tissues, which could in turn translate to reductions in shear stress. While this evidence highlights the potential therapeutic role of skin cooling for protecting tissue health, the mechanisms by which cooling enhances skin tolerance to pressure, shear, and friction remain poorly understood in humans [17-19,21-24].

In addition to its physiological effects, localized cooling of the skin can induce cold discomfort, which can limit acceptability and adherence to therapeutic interventions that promote skin integrity, particularly for vulnerable individuals at risk of pressure ulcers [25]. However, evidence on how the absolute temperature of a surface shearing against the skin contributes to thermomechanical discomfort is limited [26]. While the evidence above highlights the field’s limited mechanistic understanding of the effects of skin cooling on skin tissue viability, commercially available microclimate management systems, which deliver local and full-body cooling, continue to be developed and applied to support pressure ulcer prevention [27-29].

It is also important to note that physiological and perceptual tolerance to pressure and shear at the skin interface may also vary as a function of age and comorbidities [30], which could in turn diminish the therapeutic efficacy of cooling. For example, aging-induced changes in skin biophysics and morphology [31], as well as decreases in both reflex cutaneous vasoconstriction and density of thermoreceptors [32], are likely to modulate the thermoregulatory and perceptual sensitivity of the skin to localized cooling. Similarly, autonomic and sensory dysfunctions resulting from SCI manifest in impaired control of skin blood flow and lack of sensation below the injury level [33,34].

Based on the evidence above, this study protocol aims to investigate (1) how different levels of localized cooling influence the skin’s microvascular, inflammatory, structural, and perceptual responses to repeated pressure and shear loading at the heel; and (2) how metabolic, immunological, biophysical, and perceptual pathways underlying the effects of localized cooling on skin tolerance to shear are modulated by aging and SCI. The research that will be delivered via this protocol will be both important in improving scientific knowledge and timely to societal demands, as it will support the development of user-centered approaches to maintaining skin tissue viability via evidence-based therapeutic cooling.

## Methods

### Overview

Participants will attend 3 experimental sessions separated by a minimum of 24 hours in the Clinical Academic Facility located at Southampton General Hospital (Southampton, United Kingdom). During the sessions, participants will place the center of their posterior heel on a custom-built friction rig with an integrated thermal plate to undergo a standardized protocol eliciting repeated pressure and shear force. The study will use a randomized crossover design involving 3 plate temperatures: 36 ºC (no cooling), 24 ºC (mild cooling), or 16 ºC (strong cooling). Microclimate conditions at the interface between a loaded skin site at risk of pressure ulcer (eg, the sacrum) and a support surface (eg, a mattress) are commonly associated with skin temperatures of approximately 38 ºC. Following pilot testing, some participants (n=2) found a 38 ºC plate temperature to be borderline uncomfortable, so we decided to reduce the plate temperature to 36 ºC to improve thermal tolerability in the no cooling condition. The rationale for using 2 levels of cooling was 2-fold: first, prior research investigating localized cooling on tissue viability in animal models [17,18] used cooling interventions at 25 ºC, demonstrating the benefit of reduced skin temperature in preserving tissue viability. Second, one of the main aims of the study is to examine how different levels of cooling may influence the biophysical properties of the skin and perceptual thermal tolerances of both mild (24 ºC) and strong (16 ºC) cooling.

### Participants

Three participant cohorts will be recruited: (1) young and healthy (n =35); (2) older and healthy (n =35); and (3) with SCI (n =35). This study protocol is being supported by a Medical Research Council grant (MR/X019144/1). As part of this grant, 2 distinct experimental protocols have been designed: (1) this study protocol, which aims to investigate temperature modulation of shearing forces at the heel; and (2) our companion study protocol (Gordon et al [35]), which aims to investigate temperature modulation of sustained mechanical loading at the sacrum [35]. The separation of the protocols is motivated by their intrinsic conceptual differences (ie, the pathophysiology of shearing-induced vs loading-induced mechanical damage) as well as by different experimental challenges associated with their delivery (ie, the development of a large, shearing-inducing, and thermally controlled friction rig for the heel vs a small thermomechanical indenter for the sacrum). By design, the same participant cohort will be recruited to evaluate regional differences in responses (sacrum vs heel) and assess how the addition of shear force may impact local tissue physiology and the efficacy of cooling.

The sample size justification for this protocol is based on the power calculations from Gordon et al [35], using changes in the peak hyperemic response, which is a robust, repeatable response. Specifically, we will recruit a minimum of 18 participants per group, with target recruitment of 35 individuals, to allow for sufficient statistical power and to account for up to 50% dropout. Participants will be screened for study participation using a set of inclusion and exclusion criteria. The young and healthy participants will be aged 18 to 35 years and the older and healthy participants will be aged 55 to 70 years. The participants presenting with SCI will have an injury level within T1 to S1. The younger and older healthy groups will undertake regular physical activity (1-3 times per week), be nonsmokers and nonvapers, and be free from comorbidities that would preclude them from taking part, such as hypertension or Raynaud's disease. Both male and female participants will be recruited for a subanalysis on the influence of sex; we will also recruit individuals with different skin tones, ranging from light to dark, as assessed by the Fitzpatrick scale [36]. Stratified skin sampling will be used to determine whether there is an influence of skin tone on pressure and shear tolerance at the skin of the heel.

### Ethical Considerations

This project will recruit and test healthy individuals aged 18 to 70 years as well as individuals with SCI. The project will be conducted in line with the Southampton University Code of Practice for Research, complying with the Declaration of Helsinki. Written informed consent will be obtained from participants, as will relevant personal information (eg, perceptual skin sensitivity to cooling). Participants will have the unconditional right to withdraw from the study at any point.

Data management will adhere to the University of Southampton’ policy on data quality, which forms part of the University’s Information Governance Framework, complying with the requirements of the Data Protection Act 2018 and the University of Southampton’s Ethics Committee (Ethics and Research Governance Online; ERGO) policies. This project involves human participants and will be conducted in line with the university’s policy on the ethical conduct of research and studies involving human participants and the Medical Research Council’s policies on ethics and data sharing. Data will be fully anonymized at the earliest opportunity and before being made available with open access in the university’s data repository. All data that supports publication will be deposited and will be citable using a persistent identifier (DOI). Research findings will be published in high-quality, peer reviewed academic journals.

Participants will be eligible for compensation for their time upon successful completion of an experimental trial in the form of retail vouchers (maximum compensation value: £576 [US $785]). All experimental methods and measurements will be noninvasive, posing low risks to participants. However, mitigation measures are in place to manage any risks that may develop, including on-going skin temperature monitoring, checks of subjective well-being, and active skin rewarming following the cooling stimuli. The skin will also be checked for blanching erythema at regular intervals. Ethical approval for the stated measurements and procedures has been granted by the University of Southampton’s Ethics Committee (ERGO 88984).

### Experimental Procedures

The study uses a repeated measures cohort design. Once participants have been screened and recruited, they will be invited to their randomly allocated experimental sessions [37]. They will come to the laboratory wearing comfortable, loose-fitting attire. Upon arrival, participants will be seated while they acclimatize to the ambient conditions of the laboratory (21-24 °C; 50% relative humidity) before recording height and body mass (Seca 874; Seca GmbH).

**Figure 1:**
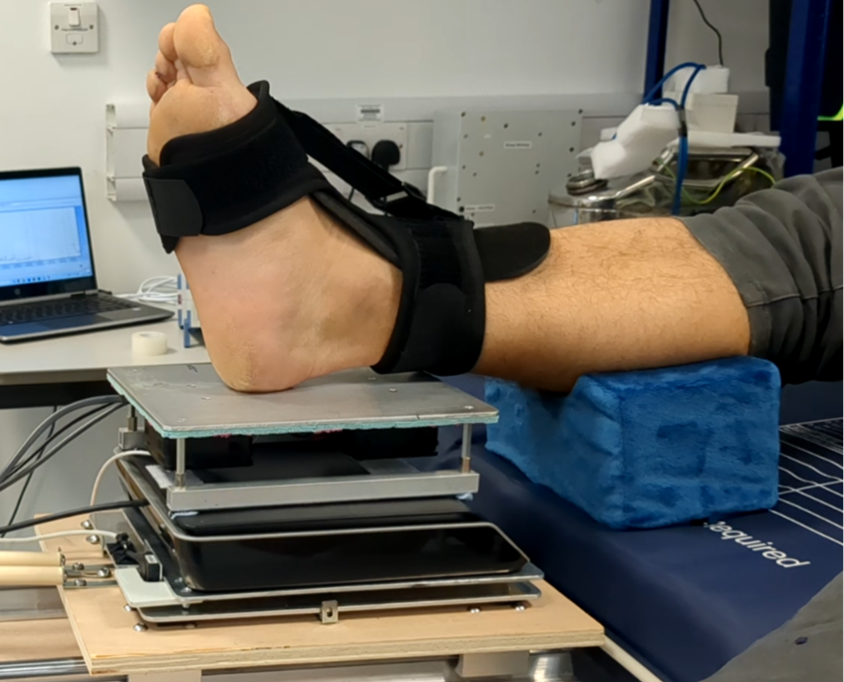
Experimental set up. The foot and ankle are supported by an orthotic brace and ankle support to standardize positioning while minimizing movement during the shearing protocol.

Following the pre-experimental checks, participants will be seated on a hospital bed in a semireclined position supported by pillows and a back rest. An elevated ankle cushion (20 × 11 × 10 cm) will then be positioned under the right leg and placed under the gastrocnemius muscle belly. To standardize the position of the foot and ankle and minimize movement during the repeated shear movements, an adjustable orthotic ankle foot brace will be strapped on to the right foot and the ankle angle set to a neutral plantar grade position (90°). This will ensure the skin over the calcaneus is consistently in contact with the surface of the thermal plate when the heel is placed on the friction rig and provide a constant relative tension in the local skin site (Figure 1). The axial load will then be standardized to exert 15 N of normal force on the thermal plate.

**Figure 2:**
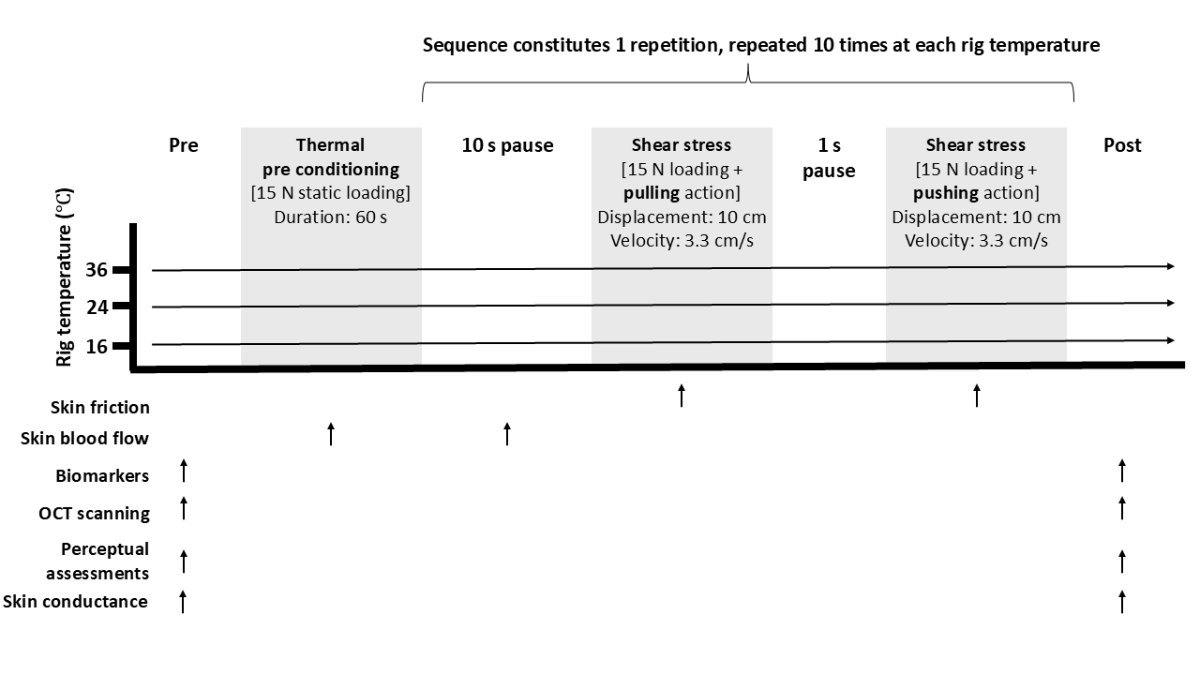
Standardized repeated shear protocol delivered at the heel. The friction rig will be used to deliver a standardized protocol to induce repeated shear stress under 3 thermal conditions: a control skin temperature with no cooling (36 °C) and 2 cooling temperatures of 24°C and 16°C. During the protocol, a series of noninvasive measurements will be conducted (estimation of skin friction from the ratio of tangential and normal fore and skin blood flow via laser Doppler flowmetry (LDF); inflammatory biomarker sampling from skin sebum; structural and functional imaging via optical coherence tomography (OCT); perceptual assessment of subjective thermal sensation, comfort, and acceptance; and measurement of skin conductance, to be used as an index of local skin hydration) at different time points (identified in the figure by the arrows).

A series of measurements that run continuously or during discrete time points will be taken (Figure 2). First, baseline assessments, including structural and functional imaging of the posterior heel skin, will be captured using optical coherence tomography (OCT). This will be followed by sampling of skin sebum at the heel for subsequent biomarker analysis, using an established methodology involving a 2-minute application time and tweezer extraction to avoid cross contamination [38]. To provide an index of baseline local skin hydration, a capacitance meter will then be placed on the heel to measure skin conductance (in microsiemens). After the baseline measurements are completed, participants will place the heel on the thermal plate of the shearing rig [13]. The temperature of the aluminum thermal plate (22 × 16.7 cm) is controlled by a set of Peltier elements, and when applied to the skin, allows for the manipulation of local skin temperature (range 0 °C to >50 °C; variable temperature rates under PID control [Daisylab; MC Computing]). An optical fiber is integrated in the center of the plate through a 1-mm hole flush to its surface, allowing for the monitoring of skin blood flow via laser Doppler flowmetry (LDF) when the heel is static. An integrated force plate and low resistance trackway allow the recording of both normal and tangential forces (in newtons), respectively. The friction rig is interfaced with an analogue-to-digital data acquisition unit (MC Computing), sampling plate surface temperature (°C), normal forces, and tangential forces at 33 Hz. The kinetic coefficient of friction (CoF) according to Amonton’s law [39] can be estimated as the ratio of tangential to normal force and defined as the resistance generated from 2 surfaces rubbing against each other [40].

The friction rig has been modified to be situated on a linear rail system that allows bidirectional linear travel. The travel is achieved by a servomotor (Drylin SAW-0630 linear module with motor; Igus), which is fixed to the rig and causes 10 cm of displacement at the heel at a velocity of approximately 3.3 cm/s. The servomotor is equipped with a linear strain gauge (Loadstar RAS1 S-Beam Load Cell; LoadStar) providing measurements on the tangential pull (in newtons) of the shearing rig during the prescribed displacements. Participants will be asked to provide subjective ratings of thermal sensation, comfort, and acceptability using Likert scales (detailed below). At this point, the standardized protocol to cause repeated shearing stress will commence (Figure 2).

The protocol starts by participants placing the heel in the center of the thermal plate over the hole, where the LDF optical probe is located flush to the surface. Positioning of the bed and lower limb will be modified to provide a standardized 15-N axial load on the plate. Skin blood flow will be measured intermittently (accounting for displacement of the heel following the static loading phases) throughout the protocol via LDF (moorVMS-LDF laser Doppler monitor; Moor Instruments). The heel will remain in place for a 60-second thermal preconditioning period to allow for temperature adaptation at the skin interface (at 36 °C, 24 °C, or 16 °C). The repeated shearing sequence will then commence (Figure 2), initially with a 10-second pause, before the servomotor travels in one direction pulling the rig and displacing it by 10 cm, pausing for 1 second, then traveling in the opposite direction, pushing the rig back to the original starting point. This sequence completes one repetition, which is repeated 10 times. After completing the 10 shearing repetitions, participants’ thermal perceptions will be recorded, followed by posttest OCT, skin sebum, and skin conductance measurements.

The sections below provide a description of the measurements to be used during the protocol.

### Measurements

#### Skin Blood Flow

Skin blood flow will be monitored via LDF using a noninvasive optical probe, sampling at a 1-mm tissue thickness [41]. LDF has been validated [42] and widely used to assess changes in blood flow velocity (as an index of changes in flow) over bony prominences, such as the sacrum [21,22,24,43]. The optical probe is integrated within the custom-built friction rig, allowing concurrent manipulation of skin temperature while monitoring changes in skin blood flow prior to the repeated shearing protocol (ie, the thermal preconditioning phase). LDF values during the thermal preconditioning phase will be used to calculate the baseline skin blood flow (taken as the mean average during the 60-second period) and subsequently compared across test shearing repetitions.

#### Biomarkers

Pro-inflammatory biomarkers (interleukin [IL]-1α, IL-1β, TNFα, IL-6, IL-8, and interferon γ [IFNγ]) and anti-inflammatory biomarkers (IL-1 receptor agonist [RA]) will be extracted from skin sebum via the application of Sebutape (Cuderm). This approach has been optimized [44] to ensure both low- and high-abundance proteins can be quantified. In summary, Sebutape is applied to the heel for 2 minutes before the samples are extracted using tweezers and a gloved hand to avoid cross contamination. Stored samples will be coded and stored at –80 °C prior to analysis using standard ELISA (enzyme-linked immunosorbent assay) plates for targeted proteins. The extraction of skin inflammatory biomarkers will use chemical and mechanical stimuli for maximal extraction efficiency. Chemical extraction will involve 0.85 mL of extraction buffer, which consists of phosphate-buffered saline + 0.1% surfactant (dodecyl maltoside). The tapes will then be shaken with the buffer for 1 hour followed by 5 minutes of sonication. A 0.35-mL aliquot will then be used for total protein analysis. The remaining 0.5 mL will be centrifuged for 10 minutes at a speed of 15,000*g* at 4 °C. The supernatants will be discarded and the remaining solution with the pellet briefly vortexed and used for the immunoassay analysis, as prescribed by the manufacturer, using MSD U-Plex kits (MesoScale Diagnostics).

#### Skin Imaging

Skin imaging will be conducted using an OCT system (VivoSight; Michelson Diagnostics Ltd) that uses a laser source of near-infrared wavelength (1305 nm; class 1M [EN 60825-1]). The system includes a dynamic mode whereby the principles of speckle variance OCT are used to visualize the microvasculature in the superficial dermis. A total of 120 images with 50-μm spacing will be acquired as a 6 × 6 × 2 mm^3^ (width × length × depth) stack. This technique will allow noninvasive characterization of the skin’s epidermal and blood perfusion properties prior to and following the thermomechanical manipulations [45]. Using OCT is noninferior to punch biopsy for skin characterization [46]. The OCT probe will be placed gently on the skin, maintaining a static position during acquisition. Spacers at the probe interface will be used to optimize the focal point of the epidermis during scanning.

#### Perceptual Assessments

Participants’ local thermal and comfort sensations will be assessed via Likert scales to establish time-dependent changes in subjective perceptions of cooling [47]. The Likert scales for thermal sensation, thermal comfort, and thermal acceptance were created based on the recommendations of Schweiker et al [48], using a ruler to draw a 100-mm horizonal line with evenly spaced anchors. Thermal sensation is measured on a 7-point scale from 1 (cold) to 7 (hot), with 4 as neutral. Thermal comfort used a 5-point scale ranging from 1 (comfortable) to 5 (extremely uncomfortable), and thermal acceptance used a 4-point scale ranging from 1 (clearly acceptable) to 4 (clearly unacceptable). Perceptual sampling will occur before and after the shear protocol (Figure 2).

### Statistical Analyses

Data will be assessed for normality of distribution with the Kolmogorov-Smirnov test. Within- and between-subject mean differences (n=35 with 95% CIs) as a function of mechanical stimulus temperature (ie, 36 ºC, 24 ºC, and 16 ºC), time (ie, varying levels depending on the variables’ sampling rate), and participant group (ie, young, older, SCI) will be analyzed. These independent and interactive effects will be assessed by means of 3-way mixed model ANOVAs (or Friedman tests) for biomarker expression, skin structural properties, biophysical properties (ie, imaging parameters), subjective thermal perceptions, and kinetic CoF. Post hoc analyses will be performed between shear stimulus temperatures, time, and participant groups based on the presence of main effects and using the Tukey test. Group-related covariables associated with sex, skin tone, and clinical status (applicable to SCI participants only) will be considered in all analyses to interpret the proportion of variance unexplained by the main effects (ie, temperature, time, and group) and their interactions.

## Results

Recruitment began in January 2024. As of February 2025, 43 participants have been enrolled in the study. Data collection and analysis are ongoing, and published findings are expected to be available in early 2026.

## Discussion

Prolonged mechanical loading from pressure, or pressure in combination with shear, over a bony prominence such as the heel can lead to skin damage and the formation of pressure ulcers [1]. Research that addresses the physiological tolerance of human skin to repeated mechanical shear is important for improving scientific knowledge and meeting societal demands. As such, this study protocol aims to investigate how cooling may influence the skin’s pathophysiological responses to repeated shear loading at the heel and how these responses may be modulated by aging and the presence of SCI. This study will provide a comprehensive understanding of the physiological processes and the potential benefits of cooling strategies to minimize the pressure ulcer risk at the posterior heel, while generating novel insights on temperature-modulated skin tolerance in vivo. This information will be relevant to skin physiologists, bioengineers, medical device manufacturers, and clinicians.

The study benefits from using a repeated measures design to purposefully sample clinically relevant groups of individuals and targeting relevant physiological and perceptual mechanisms. The heel is an anatomical location that contains minimal subcutaneous tissue, making it a vulnerable site for the development of pressure ulcers by impacting the integrity of the superficial layers of the skin [49]. The heel is particularly susceptible to injury in patients who are required to maintain semirecumbent positions, for example in bed, where individuals may repeatedly engage in repositioning (eg, from sliding down in bed) or transferring between support surfaces. Movement is often initiated by loading through the heels, creating both pressure and shear forces at the skin interface. Recent studies have shown how repeated pressure exposure, and pressure in combination with shear, elicits a biophysical and biomarker response from the skin [11,44,50,51] indicative of early signs of damage.

Previous animal data have shown that reducing skin temperature during applied mechanical loading could lower the metabolic demands of the underlying tissues, protecting metabolic and myogenic components of skin blood flow [23]. There is further evidence to indicate that lowering skin surface temperature can reduce the kinetic CoF [13]. In addition, the effects of both heat and moisture in decreasing the resilience of the epidermis in load-bearing regions of the body are well documented [52,53]. However, whether these findings from other anatomical sites translate to the skin of the heel, and how these mechanical interactions may lead to the risk of tissue damage, are unknown [10].

The study has been robustly designed but is not without limitations. Shear forces will be delivered via a dry aluminum interface, which may limit generalizability to clinical scenarios where the heel interacts with bedding with varying moisture saturation. The authors recognize the importance of evaluating the effect of temperature modulation of friction using patient-centered materials (eg, textiles used in the fabrication of chairs and beds), and the subsequent translation of clinically relevant findings. This research is currently being undertaken in our laboratory. Given the lack of data on localized cooling at the heel, identifying how the effects of skin cooling vary both physiologically and perceptually will therefore be important for developing bespoke patient-centered solutions to maintain skin integrity under shear stress that are both effective and comfortable.

## Appendix

Multimedia Appendix 1 Peer review report by the UK Research and Innovation (UKRI) Medical Research Council.

## Abbreviations

CoF: coefficient of friction
ELISA: enzyme-linked immunosorbent assay
ERGO: Ethics and Research Governance Online
IFNγ: interferon γ
IL: interleukin
LDF: laser Doppler flowmetry
OCT: optical coherence tomography
RA: receptor agonist
SCI: spinal cord injury
TNFα: tumor necrosis factor α
TRPM8: transient receptor potential melastatin 8

## Data Availability

The datasets generated or analyzed during this study are available from the corresponding author on reasonable request.
