# Supplementary material for: Repeated Pressure and Shear Stress at the Posterior Heel Following Localized Skin Cooling: Protocol for a Repeated Measures Cohort Study
Source: JMIR Res Protoc. 2025 Jul 21;14:e73250. doi: 10.2196/73250 (PMC12322604; doi:10.2196/73250)
Supplement: Multimedia Appendix 1 [file resprot_v14i1e73250_app1.pdf]

Medical Research Council
2nd Floor David Phillips Building, Polaris House, North Star Avenue, Swindon,
United Kingdom SN2 1ET
Telephone +44 (0) 1793 416200
Web https://mrc.ukri.org/

Compliance with the UK data protection legislation and the EU General Data Protection Regulations 2016/679 (GDPR)

In accordance with UK data protection legislation and the EU General Data Protection Regulations 2016/679 (GDPR), the personal data provided on this form will be processed by MRC, as part of UKRI, and may be held on computerised databases and/or manual files. Further details can be found in the **guidance notes** and on the UK Research and Innovation Privacy Notice (https://www.ukri.org/privacy-notice/).

Research Grant
Peer Review

MRC Reference: MR/X019144/1

Document Status: With Council

Experimental Medicine Full Sep 2022

Applicant Details

Applicant	Dr Davide Filingeri	Organisation	University of Southampton

Title of Research Project

Temperature modulation of skin tolerance to applied mechanical loading and shear

Review Information

Response Due Date	14/10/2022	Reviewer Reference:	026506576

Rationale

(1) Is the project underpinned by a good medical/scientific rationale? (2) Is there a reasonable body of evidence to support the proposed rationale? (3) Is the approach structured in an appropriate manner for mechanistic hypotheses to be defined and tested? (4) Is the approach likely to lead to significant new understanding of human disease mechanisms or support approaches to the detection, diagnosis, treatment or monitoring of disease?

The rationale is underpinned by our current theoretical understandanding. Skin cooling is proposed as a possible preventive intervention, but we know not much about the biological effects. The approach is structured and appropriate. One general problem is, that the loading/heating/cooling times are much shorter, compared to clinical reality. Maybe it could be slightly prololonged. The results will be very helpful to contribute to the body of evidence.

Deliverability

(1) Is the approach presented reasonable, appropriate, and justified? (2) Does the plan propose appropriate go/no-go milestones? (3) Do the applicants have the necessary expertise to deliver the plan? (4) Is there appropriate risk management for any key identified risks? (5) Is the methodology appropriate and statistically robust - are all sample sizes correctly justified and are the plans for statistical analysis appropriate for the project plan?

The presented approach is appropriate and consists of realistic workpackeges and milestones. The applications are highly qualified and experience in the described methods. The identified risks and the management are correct. The sample size

is sufficient and the statistical analysis plan appropriate. Not sure whether a primary outcome is needed here, because it is exploratory research and it is not about rejecting null hypotheses. The focus should be on interval estimation.

Ethics

Please comment on any ethical and/or research governance issues, including: (1) whether the proposed research is ethically acceptable; (2) any ethical issues that need separate consideration; (3) the appropriateness of ethical review and research governance arrangements.

The prosed research is ethically acceptable. Similar trials and skin measurements are conducted worldwide and are safe.

Resources and Management

Please comment on: (1) whether funds requested are essential and justified by the importance and scientific potential of the research; (2) investigator time and proposed involvement related to management of the research; (3) whether the proposal demonstrates value for money in terms of the resources requested; (4) whether any animal use is fully justified in terms of need, species and species sex, proposed number and conformance to guidelines.

The funds requested are needed to do this research. The scientific merits clearly outweigh the costs.

Data Management Plans

Please assess whether the data management plan indicates whether the applicants have (or are likely to have) a sound plan for managing the research data funded through the award, taking into account: (1) the types, scale and complexity of data being (or to be) managed; (2) the likely long-term value for further research including by sharing data; (3) the anticipated information security and ethical requirements.

The data management plan and information on secrurity is appropriate. The data sharing plan is transparent.

Impact

What is the potential economic and societal impact of the proposed research, including: (1) potential contributions to improvements in the understanding of disease mechanism; (2) identification of realistic potential improvements to human or population health.

Still, too many pressure ulcers develop. This indicates that current prevention strategies are not optimal. Although preventive cooling is discussed in the literature, it is not widely implemented yet. One reason might be, that our basic understandanding is insufficient. This research will help to enhance knowledge to improve pressure ulcer prevention.

Overall Evaluation

Please comment on the overall strengths and weaknesses of the proposal and its quality.

The overall strengths are:
(1) Application of many different skin measurements to get a comprehensive picture of the physiological responses.
(2) Standardized experimental condidtions. Continous blood flow measurements during loading.
(3) Large sample size.
(4) Experienced investigators.

Possible weaknesses:
(1) Too short loading times.
(2) Artificial loading/warming/cooling conditions, that are different from clinical practice.

Overall Assessment

Score 1-6

1 - Poor	2 - Good	3 - High	4 - Very High	✔ 5 - Excellent	6 - Exceptional

Medical Research Council
2nd Floor David Phillips Building, Polaris House, North Star Avenue, Swindon,
United Kingdom SN2 1ET
Telephone +44 (0) 1793 416200
Web https://mrc.ukri.org/

Compliance with the UK data protection legislation and the EU General Data Protection Regulations 2016/679 (GDPR)

In accordance with UK data protection legislation and the EU General Data Protection Regulations 2016/679 (GDPR), the personal data provided on this form will be processed by MRC, as part of UKRI, and may be held on computerised databases and/or manual files. Further details can be found in the **guidance notes** and on the UK Research and Innovation Privacy Notice (https://www.ukri.org/privacy-notice/).

Research Grant
Peer Review

MRC Reference: MR/X019144/1

Document Status: With Council

Experimental Medicine Full Sep 2022

Applicant Details

Applicant	Dr Davide Filingeri	Organisation	University of Southampton

Title of Research Project

Temperature modulation of skin tolerance to applied mechanical loading and shear

Review Information

Response Due Date	17/10/2022	Reviewer Reference:	040208585

Rationale

(1) Is the project underpinned by a good medical/scientific rationale? (2) Is there a reasonable body of evidence to support the proposed rationale? (3) Is the approach structured in an appropriate manner for mechanistic hypotheses to be defined and tested? (4) Is the approach likely to lead to significant new understanding of human disease mechanisms or support approaches to the detection, diagnosis, treatment or monitoring of disease?

(1) Is the project underpinned by a good medical/scientific rationale?
The rationale is clear as describes very well the interest and the importance of the review, with well-sustained intentions and clearly defined outcomes.
The applicant acknowledges the need to understand both the fundamentals, practices and evidence needed for the population that will be involved in the study and the contexts where it will be carried out.

(2) Is there a reasonable body of evidence to support the proposed rationale?
There is a lack of study on this subejct, but the evidence used supports really well the aim and its clearly associated with the research questions.

(3) Is the approach structured in an appropriate manner for mechanistic hypotheses to be defined and tested?
The approach / test design are mythological really interesting and are well defined.

(4) Is the approach likely to lead to significant new understanding of human disease mechanisms or support approaches to

the detection, diagnosis, treatment or monitoring of disease?
Clearly, this results will allow in the future change clinical prática or even develop medical devices to reduce the impact of the pathology

Deliverability

(1) Is the approach presented reasonable, appropriate, and justified? (2) Does the plan propose appropriate go/no-go milestones? (3) Do the applicants have the necessary expertise to deliver the plan? (4) Is there appropriate risk management for any key identified risks? (5) Is the methodology appropriate and statistically robust - are all sample sizes correctly justified and are the plans for statistical analysis appropriate for the project plan?

(1) Is the approach presented reasonable, appropriate, and justified?
The approach presentes is really well justified, with appropriated and reasonable justifications.

(2) Does the plan propose appropriate go/no-go milestones?
The 3 milestones are well described and the objectives for each milestone well thought

(3) Do the applicants have the necessary expertise to deliver the plan?
More than 15 years of experience doing this kind of research, fact that gives us security.

(4) Is there appropriate risk management for any key identified risks?
identified the risk and present solutions to minimize

(5) Is the methodology appropriate and statistically robust - are all sample sizes correctly justified and are the plans for statistical analysis appropriate for the project plan?
The methodology is robust and the statistical analysis is well described and respondes to the hypotheses defined by the group

Ethics

Please comment on any ethical and/or research governance issues, including: (1) whether the proposed research is ethically acceptable; (2) any ethical issues that need separate consideration; (3) the appropriateness of ethical review and research governance arrangements.

(1) whether the proposed research is ethically acceptable;
The risks and limitations in the study are well described. They have already the authorization forth Ethical Committee for the study - Healthy volunteers (milestone 1)

(2) any ethical issues that need separate consideration;
They have tho have a separate ethical approval for the SCI Group, because this is a vulnerable group with specific characteristics. They wrote on the submission "We will seek a protocol modification University of Southampton Ethics Committee (ERGOII) to incorporate testing of the SCI cohorts within the initial 2 months."

(3) the appropriateness of ethical review and research governance arrangements.
Appropriated, Its take in consideration the need to have a authorization to the SCI Group

Resources and Management

Please comment on: (1) whether funds requested are essential and justified by the importance and scientific potential of the research; (2) investigator time and proposed involvement related to management of the research; (3) whether the proposal demonstrates value for money in terms of the resources requested; (4) whether any animal use is fully justified in terms of need, species and species sex, proposed number and conformance to guidelines.

The item 5,2 describes clearly well the importance of the material to be used, including the investigator time and activities during the research.
The resources need for dissemination, pay the traves of the volunteers its all well defines
No animals are involved in the study!

Data Management Plans

Please assess whether the data management plan indicates whether the applicants have (or are likely to have) a sound plan for managing the research data funded through the award, taking into account: (1) the types, scale and complexity of data being (or to be) managed; (2) the likely long-term value for further research including by sharing data; (3) the anticipated information security and ethical requirements.

The applicants state that the project complies with the requirements of the Data Protection Act 2018 (DPA) and University of Southampton Ethics Committee (ERGO) policies.

The responsibility of the data management, storage and curated is from the secure Southampton PURE file space.

it's also stated that the University provides secure group-workspaces via University's networked storage via Research File store that will allow the PI, RA, CoI and collaborators to securely share work files.

Impact

What is the potential economic and societal impact of the proposed research, including: (1) potential contributions to improvements in the understanding of disease mechanism; (2) identification of realistic potential improvements to human or population health.

The findings of the research will generate novel insights on temperature-modulated skin tolerance in vivo, which will be relevant to researchers and Health professionals.

The outcomes will be relevant, as the applicants describe for skin physiologists, bioengineers, and clinicians such as dermatologists and intensive care nurses, to better understand the physiological processes and the potential benefits of cooling strategies to minimise the individual PU risk in a clinical setting.

This results will create a major impact in the changes of clinical practice and a new line of research to medical devices, increasing the knowledge of strategies to prevent pressure ulcers

Overall Evaluation

Please comment on the overall strengths and weaknesses of the proposal and its quality.

The proposal is really interesting and has a huge impact to the future of pressure ulcer prevention
The strength is Aldo the uniqueness of the study in a area were is a lack of knowledge and comprehension

The weakness are more related with difficulties in the recruitment of healthy volunteers (but they will minimize regarding the networking and experience), the recruitment of SCI will not be easy to get.

The last is related with variability of the results, healthy volunteers and the SCI group. Probably this study will continue, trying to have bigger samples to have more robust findings

Outstanding project of research. Congrats

Overall Assessment

Score 1-6

1 - Poor	2 - Good	3 - High	4 - Very High	5 - Excellent	✔ 6 - Exceptional

Medical Research Council
2nd Floor David Phillips Building, Polaris House, North Star Avenue, Swindon,
United Kingdom SN2 1ET
Telephone +44 (0) 1793 416200
Web https://mrc.ukri.org/

Compliance with the UK data protection legislation and the EU General Data Protection Regulations 2016/679 (GDPR)

In accordance with UK data protection legislation and the EU General Data Protection Regulations 2016/679 (GDPR), the personal data provided on this form will be processed by MRC, as part of UKRI, and may be held on computerised databases and/or manual files. Further details can be found in the **guidance notes** and on the UK Research and Innovation Privacy Notice (https://www.ukri.org/privacy-notice/).

Research Grant Peer Review

MRC Reference: MR/X019144/1

Document Status: With Council

Experimental Medicine Full Sep 2022

Applicant Details

Applicant	Dr Davide Filingeri	Organisation	University of Southampton

Title of Research Project

Temperature modulation of skin tolerance to applied mechanical loading and shear

Review Information

Response Due Date	07/11/2022	Reviewer Reference:	189563809

Rationale

(1) Is the project underpinned by a good medical/scientific rationale? (2) Is there a reasonable body of evidence to support the proposed rationale? (3) Is the approach structured in an appropriate manner for mechanistic hypotheses to be defined and tested? (4) Is the approach likely to lead to significant new understanding of human disease mechanisms or support approaches to the detection, diagnosis, treatment or monitoring of disease?

Certainly there is strong clinical relevance for this research due to the challenges from pressure ulcers in clinical situations (e.g., bedridden, wheelchair design, wearing of oxygen and other masks). This makes it relevant across a wide range of both patients and health care providers.

There is a reasonable body of evidence to support the proposed rationale. Skin properties are certainly affected by local environmental conditions (e.g., temperature, humidity). This is at the very heart of the mechanisms for pressure ulcers, immersion foot, etc. And shear force, along with the risk factors of higher skin temperatures, are the main contributors to blister development.

It may be due to logistical constraints, but the choice of the heel for Exp2 (shear forces) seems a strange one, given that it is by far the thickest on the entire body. With such a thick epidermal layer, it would be much less affected by any thermal changes across all parameters (skin blood flow, hydration, biomarkers, etc.). It also would not seem to translate well to the intended clinical sites (e.g., face, back, buttocks, etc.). Would it be more representative to target a site such as the forearm (non-glabrous) or palm/finger (glabrous), especially since there is a very large body of research on skin blood flow at these

sites? Or alternately, have the participants be in a prone position and study shear forces also on the sacral area to maintain consistency in studied skin area?

It seems a strange choice also to target the heel for Exp2 given the target participant on SCI individuals, where the altered neural and vascular dynamics to the heel would be very non-representative of where SCI individuals would be most at risk for pressure ulcers (e.g., buttocks and hamstrings from sitting). Overall it seems the choice of heel for Exp2 greatly limits the study.

The proposed studies by themselves will likely provide direct new medical products by itself, but will provide important insight into the relationship between skin temperature, skin blood flow, pressure, and shear forces that can be used to develop products or health guidelines.

Would modeling the effects of pressure be improved with different pressure levels rather than the proposed on (60 mmHg) / off (0.4 mmHg) design? There doesn't seem the possibility for an actual dose response of pressure currently.

Deliverability

(1) Is the approach presented reasonable, appropriate, and justified? (2) Does the plan propose appropriate go/no-go milestones? (3) Do the applicants have the necessary expertise to deliver the plan? (4) Is there appropriate risk management for any key identified risks? (5) Is the methodology appropriate and statistically robust - are all sample sizes correctly justified and are the plans for statistical analysis appropriate for the project plan?

1. The approach appears reasonable, appropriate, and justified. There seems a reasoned and reasonable timeline, personnel plan, institutional support, etc.

2. Difficult to assess. In 5.1 there are phased milestones presented with clear goals for the milestones and reasons why they are achievable, but not necessarily any plans in case those milestones are not achieved.

3. Yes, there is complementary expertise amongst the research team. The PI is a leader in the field of skin physiology in terms of how it senses thermal and wetness stimuli, and the two CIs bring expertise in skin health, backed by an established Skin Health Centre at U Southhampton. There is a track record in recruiting from the desired participant demographic.

4. The risks to successful completion are clearly laid out and this is generally the case also with mitigation strategies.

5. See comments in "Rationale" about specific methodological issues. The statistical plan appears robust.

Ethics

Please comment on any ethical and/or research governance issues, including: (1) whether the proposed research is ethically acceptable; (2) any ethical issues that need separate consideration; (3) the appropriateness of ethical review and research governance arrangements.

The ethical risk to participants is minimal and adequately argued. The procedures for ethical review and research governance are appropriate.

Resources and Management

Please comment on: (1) whether funds requested are essential and justified by the importance and scientific potential of the research; (2) investigator time and proposed involvement related to management of the research; (3) whether the proposal demonstrates value for money in terms of the resources requested; (4) whether any animal use is fully justified in terms of need, species and species sex, proposed number and conformance to guidelines.

Funding request appears essential and appropriate, although not being from UK I'm not fully familiar with what is typically requested within such grants.

No animal work is being proposed.

Data Management Plans

Please assess whether the data management plan indicates whether the applicants have (or are likely to have) a sound plan for managing the research data funded through the award, taking into account: (1) the types, scale and complexity of data being (or to be) managed; (2) the likely long-term value for further research including by sharing data; (3) the anticipated information security and ethical requirements.

Strong part of proposal is the eventual release of data as open access and shared across interested researchers. This is coupled by planned workshops in years 2 and years 3 to disseminate research with interested clinical and industrial partners.

Impact

What is the potential economic and societal impact of the proposed research, including: (1) potential contributions to improvements in the understanding of disease mechanism; (2) identification of realistic potential improvements to human or population health.

1. Study doesn't provide new insight into disease mechanism, as the risks for pressure ulcers and skin health are already known and not the direct focus of this proposal. Rather, this proposal focuses on a potential countermeasure to mitigate the prevalence of pressure ulcers. This is not a negative to the proposal, but rather placing it in proper context.

2. This proposal will not likely provide immediate countermeasures against pressure ulcers, but the information can highlight a potential pathway towards reducing its prevalence that can be ultimately translated to clinical tools (e.g., equipment) and guidelines.

Overall Evaluation

Please comment on the overall strengths and weaknesses of the proposal and its quality.

The proposal can lead to eventual countermeasures that reduce the risks for pressure ulcers. Specifically, if it is demonstrated through this proposal that skin cooling can reduce skin pressure and improve blood flow, it can lead to developing tools (e.g., cooling mattresses or wheelchair seating) or guidelines to reduce the risk of pressure ulcers. Therefore, I would assess the proposal as important and relevant to MRC.

The main and important hesitation I have concerns using the heel as the test site for Exp2. I just don't think the very thick skin of the heel is representative of any other skin site on the body. I would test the same sacral surface as in Exp1 for both ecological validity and also for easier comparison with findings from Exp1.

The team itself is strong and appears to have all relevant research expertise and institutional resources/support to successfully execute the proposal.

Overall Assessment

Score 1-6

1 - Poor	2 - Good	3 - High	✔ 4 - Very High	5 - Excellent	6 - Exceptional
